# Supplementary material for: Transcriptional regulation of autophagy-lysosomal function in BRAF-driven melanoma progression and chemoresistance
Source: Nat Commun. 2019 Apr 12;10:1693. doi: 10.1038/s41467-019-09634-8 (PMC6461621; doi:10.1038/s41467-019-09634-8)
Supplement: Supplementary file 1 — Supplementary Information [file 41467_2019_9634_MOESM1_ESM.pdf]

Supplementary information

Transcriptional regulation of autophagy-lysosomal function in BRAF-driven melanoma progression and chemoresistance

Li, et al.

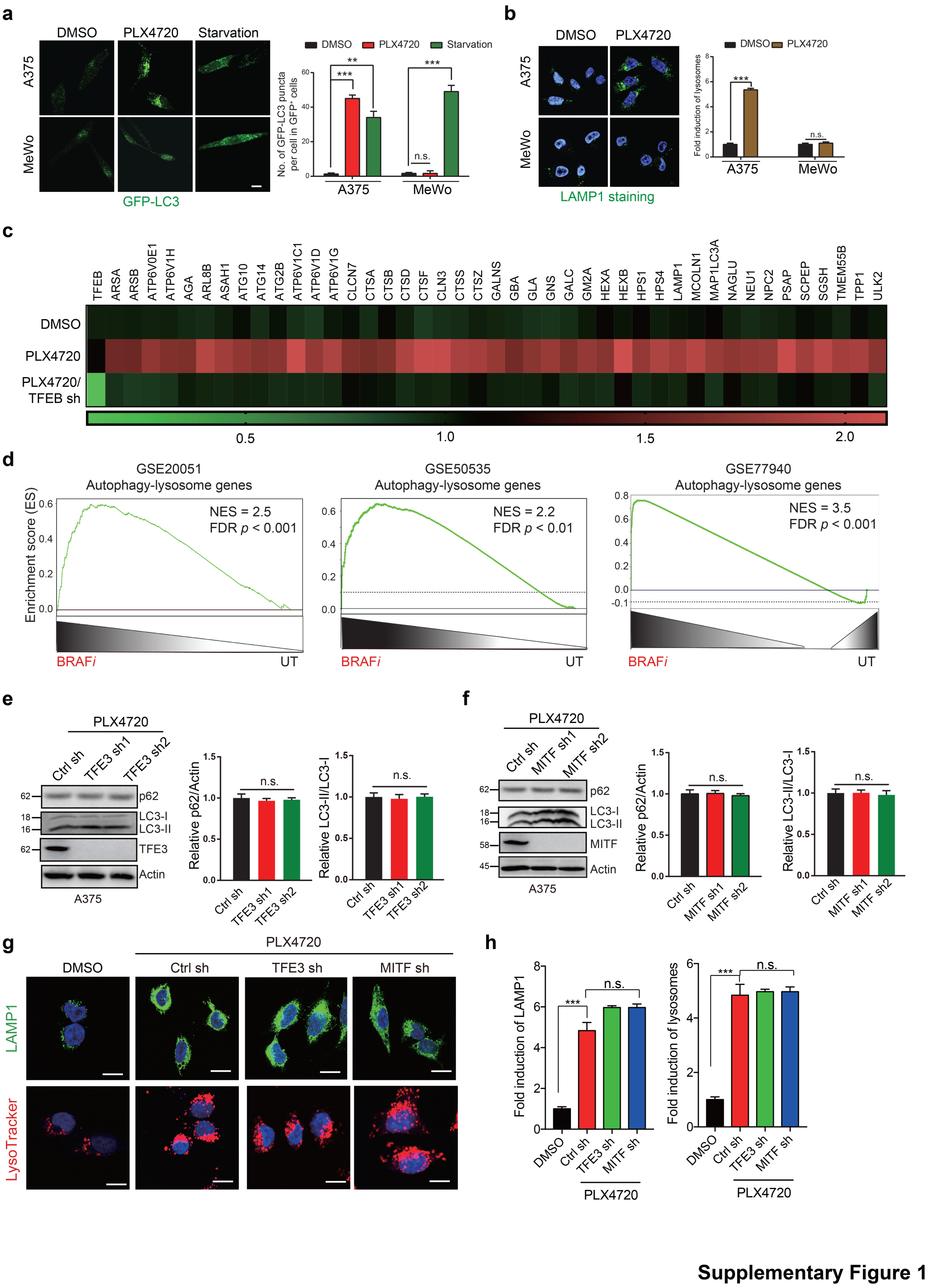

Supplementary Figure 1. PLX4720 induces autophagy-lysosomal activation through TFEB.

a Representative images of GFP-LC3 puncta in A375 and MeWo human melanoma cells treated with DMSO, PLX4720 (1 μM, 12 h), or starvation (6 h). Quantifications of GFP-LC3 puncta per cell of GFP-positive cells are shown (right panel). *n* = 3 independent experiments.
b Representative images of immunostaining for LAMP1 (lysosome marker) in A375 and MeWo cells treated for 12 h with DMSO or PLX4720 (1 μM). Quantifications of the relative fold induction of lysosomes by PLX4720 are shown (right). *n* = 3 independent experiments.
c Upregulation of the autophagy-lysosomal genes in PLX4720 (1 μM, 12 h)-treated A375 human melanoma cells as compared to the vehicle control, which is inhibited upon TFEB depletion. Upregulation is indicated in red, downregulation is indicated in green, and a similar expression is indicated in black, as generated by GraphPad Prism 7.
d GSEA of different human melanoma data sets (GSE20051[1], GSE50535[2], GSE77940[3]) for enrichment of the autophagy-lysosomal gene signature after administration of BRAF(V600E) inhibitors (Gene Expression Omnibus (GEO) accession numbers indicated). UT, untreated. FDR, false-discovery rate; NES, normalized enrichment score.
e,f Western blot analysis (left) and densitometric quantification (right) of the LC3-II/LC3-I and p62/Actin ratios in A375 cells treated with shRNA-mediated knockdown of TFE3 (**e**) or MITF (**f**). Actin served as a loading control. sh, shRNA. *n* = 4 independent experiments.
g,h Representative images (**g**) and quantification (**h**) of the LAMP1 (green) immunostaining and LysoTracker Red in PLX4720 (1 μM, 12 h)-treated A375 cells with the depletion of TFE3 or MITF. *n* = 3 independent experiments.

Scale bars, 10 μm. For all quantification, data represents the mean ± SD derived from the indicated number of independent experiments. Comparisons were made using Student's *t* test. **, $P < 0.01$; ***, $P < 0.001$; n.s., not significant.

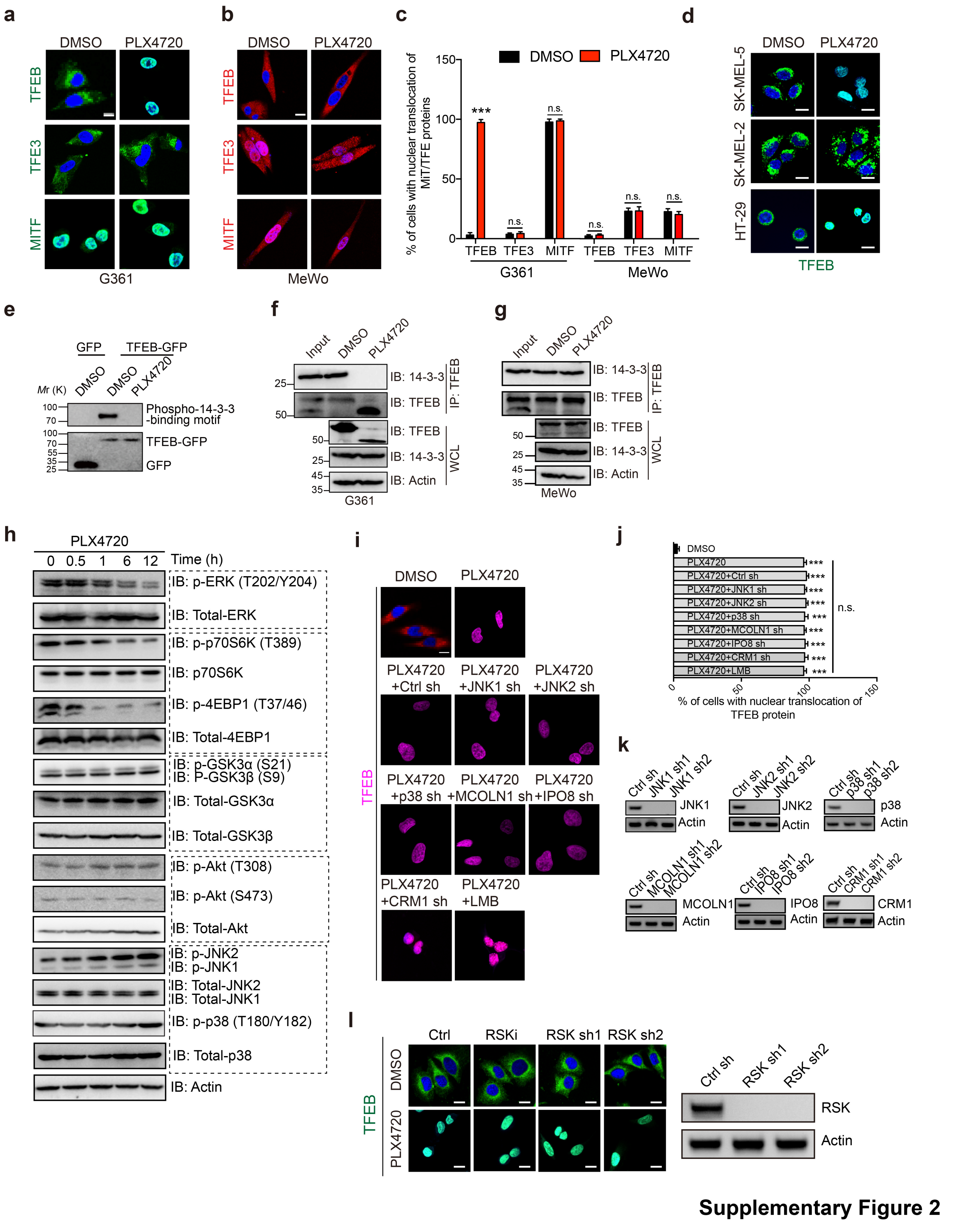

Supplementary Figure 2

Supplementary Figure 2. PLX4720 induces TFEB-mediated autophagy-lysosomal activation by ERK inhibition.

a-c Confocal microscopy analyses of the subcellular distribution of endogenous TFEB, TFE3, and MITF in PLX4720 (1 μM, 12 h)-treated G361 cells (**a**) and MeWo cells (**b**). The nuclear translocation of endogenous TFEB, TFE3, and MITF in cells shown in (**a**) and (**b**) was quantified in (**c**). n = 150 cells obtained by gathering data of three independent experiments.
d Confocal microscopy analyses of the subcellular distribution of endogenous TFEB in PLX4720 (1 μM, 12 h)-treated SK-MEL-5 ($BRAF^{V600E}$-positive) and SK-MEL-2 ($NRAS^{Q61R}$, $BRAF^{WT}$) melanoma cells, and HT29 ($BRAF^{V600E}$-positive) colon cancer cells. Representative images of three independent experiments were shown.
e Immunoblot analysis of the phosphor-14-3-3-binding motif (upper) of TFEB-GFP precipitated with GFP-Trap beads (lower) in A375 cells treated with DMSO or PLX4720 (1 μM, 12 h).
f,g Endogenous TFEB interaction with 14-3-3 proteins in PLX4720 (1 μM, 12 h)-treated G361 cells (**f**) and in MeWo cells (**g**). Data are from one experiment that is representative of three independent experiments.
h Effect of PLX4720 on the kinase activities that have been involved in TFEB regulation. Whole cell lysates (WCLs) of A375 cells treated with PLX4720 (1 μM) for the indicated time were used for immunoblotting (IB) with the indicated antibodies. Note the reduced levels of phosphorylated ERK, S6K, and 4E-BP1 and increased levels of JNK1/2 and p38 in response to PLX4720 as indicated. Actin served as a loading control.
i-k Representative confocal images (**i**) and quantification (**j**) of nuclear localization of endogenous TFEB (purple) in PLX4720 (1 μM, 12 h)-treated A375 cells upon the shRNA-mediated depletion of JNK1, JNK2, p38, MCOLN1, IPO8, or CRM1 or upon leptomycin B (LMB; 20 nM, 2 h) treatment. Western blot analysis shows endogenous protein expression in cells in (**k**). $n =$ 4 independent experiments.
l Representative confocal images (left) of nuclear localization of endogenous TFEB (green) in PLX4720 (1 μM, 12 h)-treated A375 cells upon knockdown of RSK by two different shRNA (sh1 and sh2) or upon RSK inhibition by RSK inhibitor (RSKi) SL0110 (50 μM, 24 h). Expression of RSK in indicated A375 cells is shown by western blot analysis (right). Actin served as a loading control.

Scale bars, 10 μm. Data in e, f, g, h, k, and l are from one experiment that is representative of three independent experiments. For all quantification, data represents mean ± SD derived from indicated number of independent experiments. Comparisons were made using Student's *t* test. ***, $P < 0.001$; n.s., not significant. See Supplementary Fig. 13 for uncropped data of **e** and **h**.

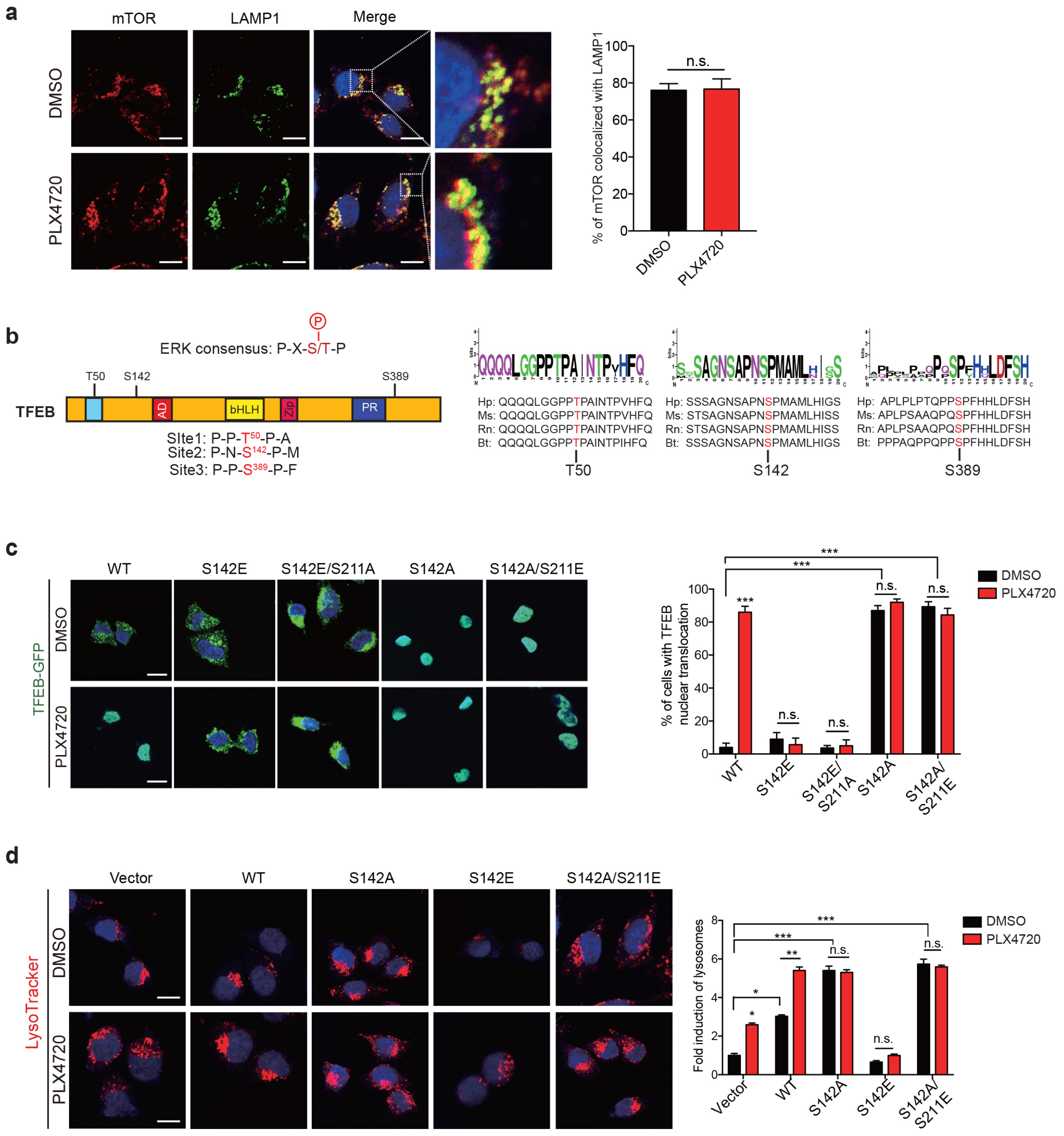

Supplementary Figure 3

Supplementary Figure 3. The predominant role of TFEB S142 phosphorylation by ERK in TFEB regulation and autophagy-lysosome activation in BRAF-mutant melanoma.

a PLX4720 treatment does not affect mTOR lysosomal association. Representative images (left) and quantification (right) of endogenous mTOR (red) and LAMP1 (green) staining in A375 cells treated without or with PLX4720 (1 μM, 12 h). *n* = 3 independent experiments.

b The consensus ERK phosphorylation motif and candidate ERK phosphorylation sites in human TFEB. X represents any amino acid.
c Subcellular distribution (left) and quantification (right) of the nuclear localization of TFEB-GFP with the indicated point mutations in A375 cells. *n* = 3 independent experiments.
d Representative images (left) and quantification (right) of LysoTracker Red (red) staining of PLX4720 (1 μM, 12 h)-treated A375 cells stably expressing WT or mutant TFEB as indicated. *n* = 3 independent experiments.

Scale bars, 10 μm. For all quantification, data represents the mean ± SD derived from indicated number of independent experiments. Comparisons were made using Student's *t* test. *, $P < 0.05$; **, $P < 0.01$; ***, $P < 0.001$; n.s., not significant.

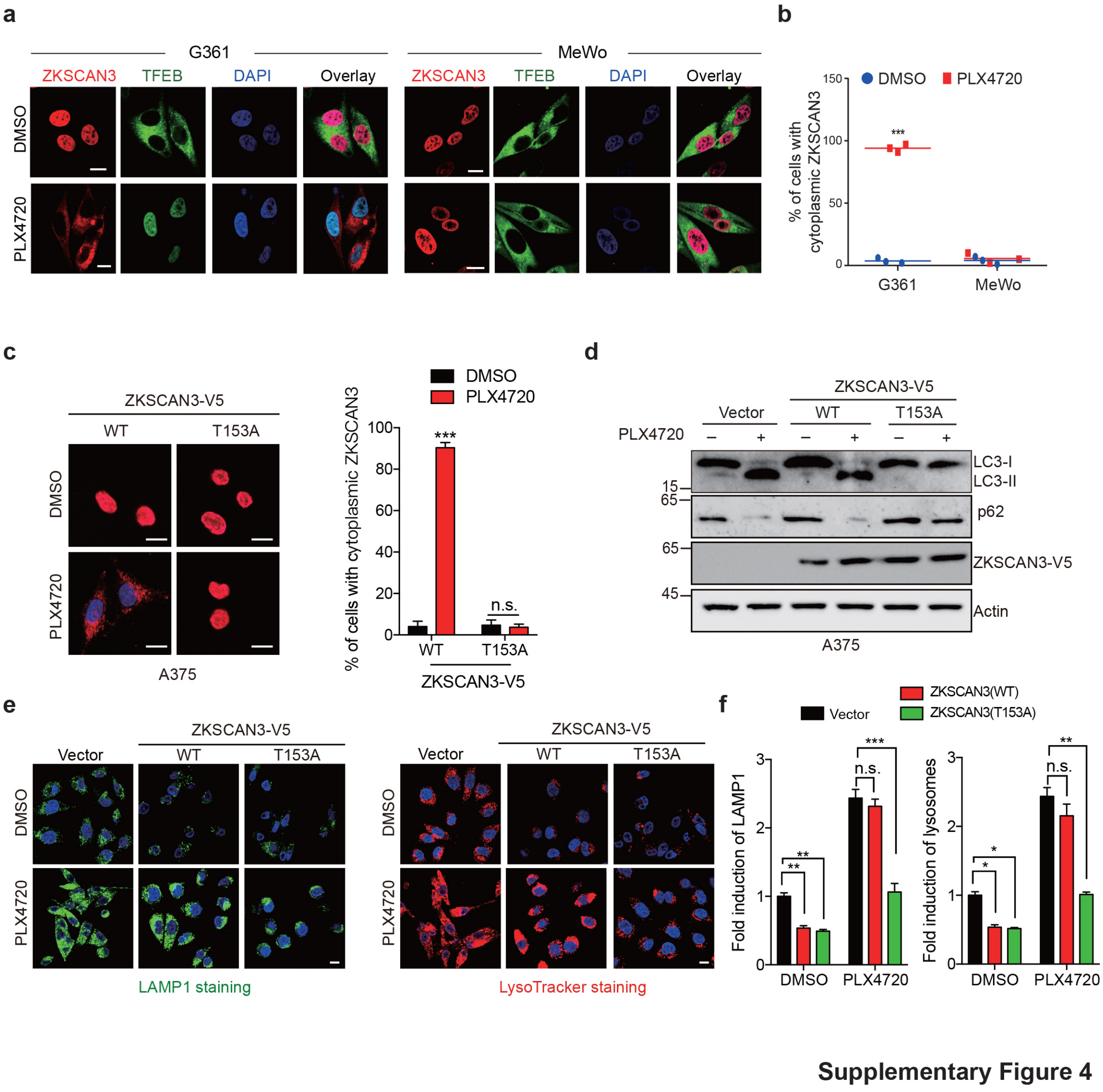

Supplementary Figure 4. PLX4720 induces ZKSCAN3 T153 phosphorylation and cytoplasmic translocation in BRAF^{V600E} melanoma cells.

a Representative confocal images of subcellular translocation of endogenous TFEB (green) and ZKSCAN3 (red) in G361 cells (left) and MeWo cells (right) treated with DMSO or PLX4720 (1 μM, 12 h). The nucleus was stained by DAPI (blue). $n =$ 5 independent experiments.

b Quantification of ZKSCAN3 cytoplasmic translocation in cells shown in (**a**).

c ZKSCAN3 T153A mutation ablated the effect of PLX4720 (1 μM, 12 h) in inducing cytoplasmic translocation of ZKSCAN3. Representative images (left) and quantification (right) of ZKSCAN3 proteins as indicated are shown. $n =$ 3 independent experiments.

d Western blot analysis of p62 and LC3-II/LC3-I in A375 cells expressing WT or mutant ZKSCAN3 in the presence or absence of PLX4720 (1 μM, 12 h) treatment. Data are from one

experiment that is representative of three independent experiments. See Supplementary Fig. 13 for uncropped data.

e Enforced expression of ZKSCAN3(T153A) antagonizes PLX4720-induced lysosome biogenesis. A375 cells expressing empty vector, WT ZKSCAN3, or T153A ZKSCAN3 mutant were treated with PLX4720 (1 μM, 12 h) and stained with LAMP1 (left) or LysoTracker Red (right).

f Quantification of the LAMP1 staining of cells shown in (e). $n = 3$ independent experiments.

Scale bars, 10 μm. For all quantification, data represents the mean ± SD derived from indicated number of independent experiments. Comparisons were made using Student's t test. *, $P < 0.05$; **, $P < 0.01$; ***, $P < 0.001$; n.s., not significant.

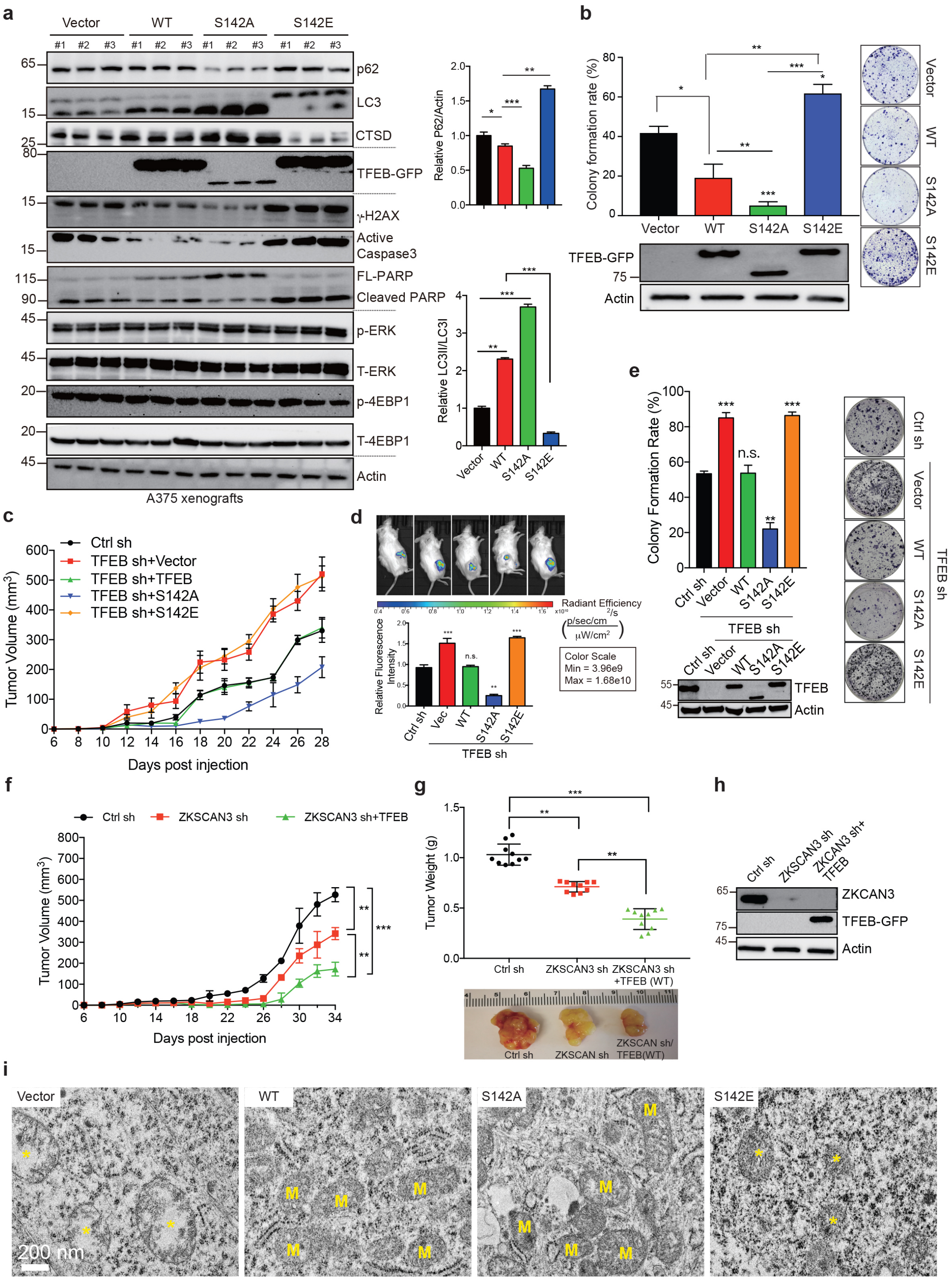

Supplementary Figure 5

Supplementary Figure 5. Effect of TFEB and ZKSCAN3 on $BRAF^{V600E}$-driven melanoma progression.

a Western blot analysis of autophagy (p62 and LC3), lysosome (Cathepsin D, CTSD), DNA damage (γ-H2AX), apoptosis (active Caspase 3 and PARP cleavage), ERK activity (p-ERK), and mTOR activity (p-4EBP1) in the indicated xenograft tumor genotypes (three randomly chosen samples per group; similar results observed in all 10-12 samples per group). Densitometric quantifications of the p62/Actin and LC3-II/LC3-I ratios in the indicated xenografts are shown (right panel). $n = 3$ independent experiments. FL-PARP, full-length PARP.

b Colonogenic survival of A375 melanoma cells stably expressing vector, WT TFEB, S142A or S142E TFEB mutant. Bars are the mean ± SD percentage of colonies for each group after 14 days. Representative images of colony-forming ability are shown (right panel); western blots show the protein expression as indicated (bottom panel). $n = 3$ independent experiments.

c Tumor volume of xenografts formed after subcutaneous injection of NOD/SCID mice with TFEB knockdown (TFEB sh) A375 cells reconstituted with vector, WT or mutant TFEB as indicated. Control shRNA (pGIPZ)-transfected cells were injected in parallel. Results are the mean volume ± SD for 5-6 mice per group per time point.

d Bioluminescence images (top) of tumor volume of the indicated A375 xenograft tumor genotype in live NOD/SCID mice at Day 28 after inoculation. Radiant efficiency expressed as $p/sec/cm^2/sr/(\mu W/cm^2)$ was quantified (bottom).

e Colonogenic survival of TFEB knockdown A375 melanoma cells reconstituted with vector, WT or mutant TFEB as indicated. Representative images of colony-forming ability are shown (right panel); western blots show TFEB protein expression (bottom). $n = 3$ independent experiments.

f Depletion of ZKCAN3 synergizes with TFEB overexpression in suppressing $BRAF^{V600E}$-driven tumor progression. Tumor volume of xenografts formed after subcutaneous injection of NOD/SCID mice with A375 cells stably expression ZKSCAN3-specific shRNA with or without ectopic expression of TFEB(WT). Results are the mean volume ± SD for 10-12 xenografts per group per time point.

g Tumor weights from experiment in (f) upon autopsy at Day 34. Results are the mean weight (g) ± SD for 10-12 xenografts per group per time point.

h Western blot analysis of the indicated protein expression in A375 cells from experiment in (f). $n = 3$ independent experiments.

i Representative EM images of A375 xenografts expressing vector, WT TFEB, or S142A or S142E TFEB mutant. Note that more swollen/damaged mitochondria were detected in vector- and $TFEB^{S142E}$-expressing A375 cells. M, normal mitochondria. Asterisks denote swollen or damaged mitochondria.

For all quantification, data represents the mean ± SD from the indicated number of independent experiments. Comparisons were made using Student's *t* test. *, $P < 0.05$; **, $P < 0.01$; ***, $P < 0.001$; n.s., not significant. See Supplementary Fig. 13 for uncropped data of **a**, **b**, **e**.

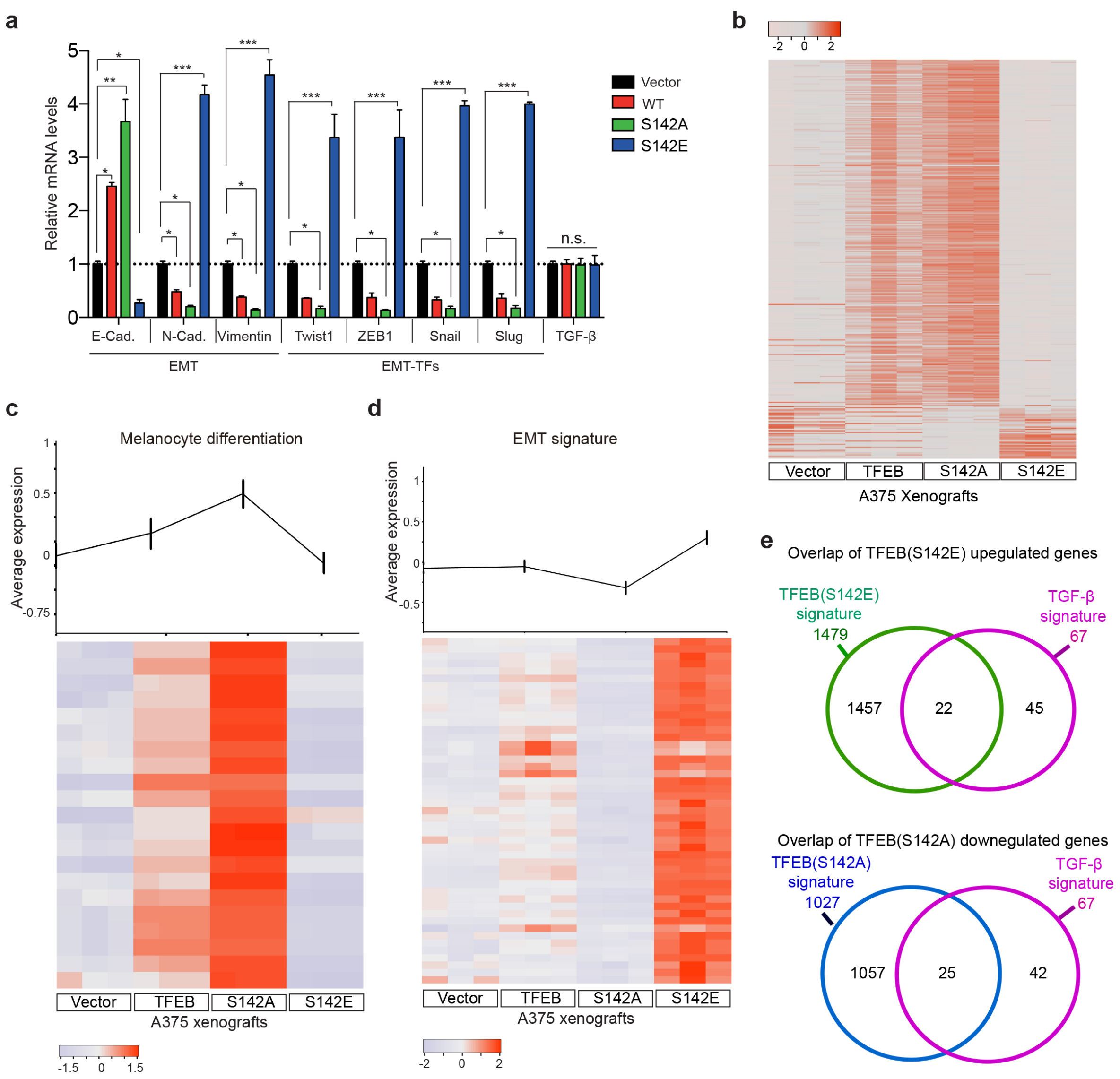

Supplementary Figure 6

Supplementary Figure 6. Effect of TFEB on the transcriptome of BRAF^{V600E} melanoma.

a Quantitative RT-PCR showing the relative levels of mRNA expression for EMT-related molecules in indicated xenograft tumor genotype. Data are the mean ± SD from three independent experiments.

b A heat map showing 375 most differentially expressed genes (FDR $P < 0.05$) in the indicated xenograft tumor genotypes (three replicates per group). Note that the expression profile of the vector and S142E tumor samples showed a closely similar pattern that is distinct from the pattern of WT and S142A mutant tumor samples.

c One dimensional hierarchical clustering of differentially expressed genes (FDR $P < 0.05$) between S142A and S142E TFEB mutant tumor samples involved in melanocyte differentiation. Also refer to **Supplementary Data 1b.**
d One dimensional hierarchical clustering of genes involved in EMT transdifferentiation (FDR $P < 0.05$) that were significantly upregulated by expression of $TFEB^{S142E}$. Also refer to **Supplementary Data 1c.**
e Venn diagrams showing the number of differentially expressed signature genes downregulated in $TFEB^{S142A}$ and upregulated in $TFEB^{S142E}$ tumor samples (FDR $P < 0.05$) over the vector control group that overlapped with the genes involved in the TGF-β signaling pathway. Also refer to **Supplementary Data 1e,f**.

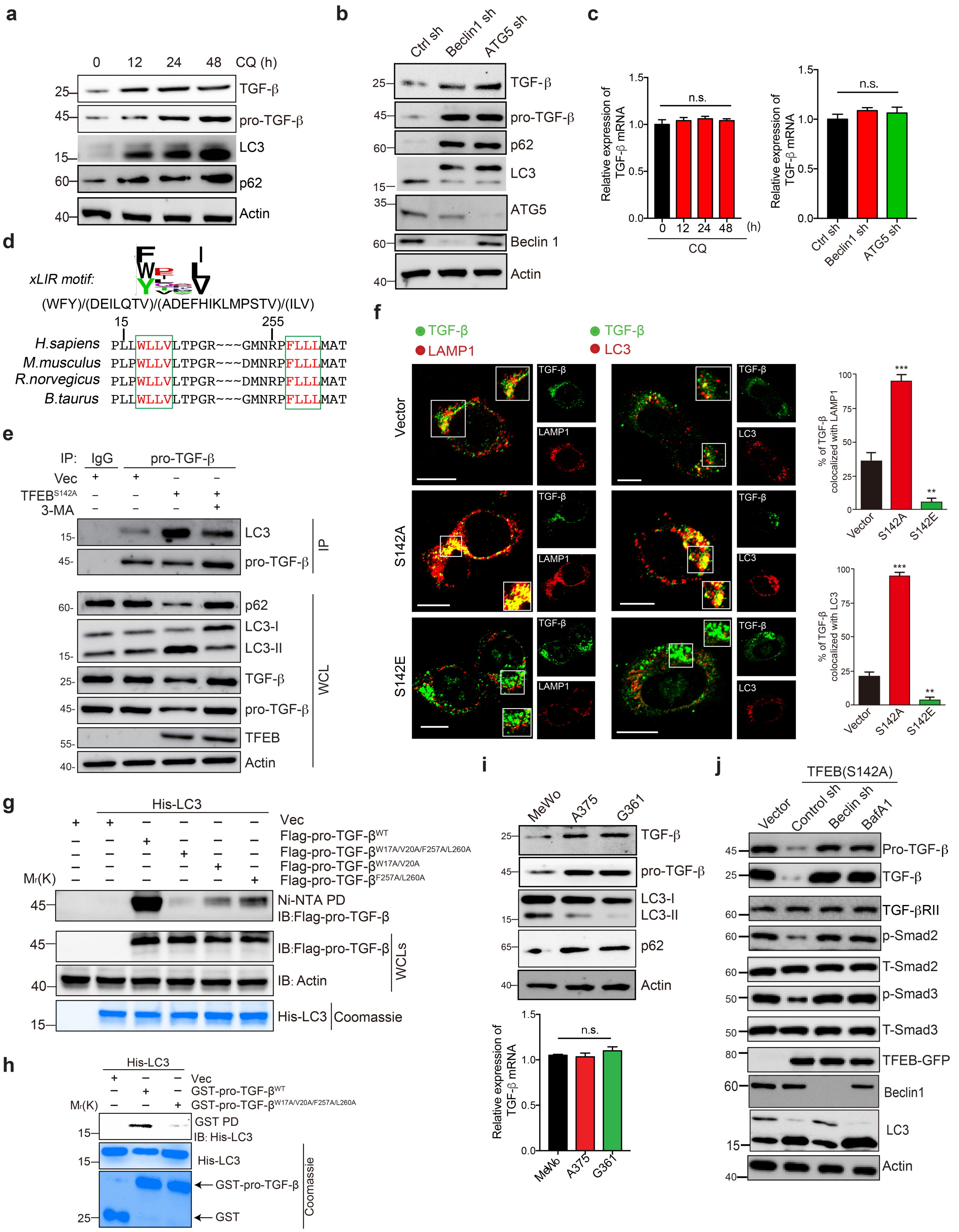

Supplementary Figure 7

Supplementary Figure 7. TFEB-mediated autophagy-lysosomal function on the protein turnover of TGF-β.

a,b Western blot analysis of TGF-β proteins (pro-TGF-β and mature TGF-β) and autophagy markers (p62 and LC3I/II) in A375 cells treated with chloroquine(CQ; 50 μM) for indicated time (**a**) or with shRNA-mediated depletion of ATG5 and Beclin1 (**b**). Actin serves as a loading control.

c Quantitative RT-PCR analysis of TGF-β mRNA expression in cells in (a) and (b). *n* = 3 independent experiments.

d The consensus LC3-interacting region (xLIR) motif (ADEFGLPRSK)(DEGMSTV)(**WFY**)(DEILQTV)(ADEFHIKLMPSTV)(**ILV**) in human TGF-β, which is conserved across species.

e Expression of $TFEB^{S142A}$ promotes interaction between endogenous pro-TGF-β and LC3 in A375 cells, which is inhibited by 3-MA. WCLs of A375 cells stable expressing vector or $TFEB^{S142A}$ in the presence/absence of 3-MA (100 nM, 24 h) treatment were used for IP with control IgG (lane 1) or anti-pro-TGF-β antibody, followed by IB with the indicated antibodies. The bottom panels showed indicated protein expression with actin as a loading control. Note the correlation of increased autophagy by $TFEB^{S142A}$ with decreased pro-TGF-β and increased pro-TGF-β association with LC3.

f Subcellular distribution of TGF-β (green) relative to the lysosomes (red) or to the LC3-labelled autophagosomes (red) in A375 cells expressing empty vector, $TFEB^{S142A}$ or $TFEB^{S142E}$. The percentage of TGF-β associated with the autophagy and lysosomal compartments are quantified (right panels). *n* = 200 cells pooled from three independent experiments.

g Pulldown (PD) assays using recombinant His-LC3 and lysates from A375 cells expressing Flag-tagged WT pro-TGF-β or W17A/V20A, F257A/L260A, W17A/V20A/F257A/L260A pro-TGF-β mutants. Note signficantly reduced LC3 binding with pro-TGF-β mutants. Actin serves as a loading control. Input recombinant proteins visualized with Coomassie blue staining are shown (lower panel).

h Recombinant His-LC3 interacts with recombinant GST-tagged WT pro-TGF-β but not its W17A/V20A/F257A/L260A mutant. Lower panels show input recombinant proteins by Coomassie blue staining.

i Western blot analysis of TGF-β proteins (pro-TGF-β and mature TGF-β) and autophagy markers (p62 and LC3I/II) in $BRAF^{WT}$ (MeWo) and $BRAF^{V600E}$ mutant (A375 and G361) melanoma cells. Quantitative RT-PCR analysis of pro-TGF-β mRNA expression in these cells is also shown (bottom).

j Inhibition of the autophagy and lysosomal function abolished the effect of $TFEB^{S142A}$ on suppression of TGF-β levels and TGF-β signaling. A375 cells stably expressing $TFEB^{S142A}$ were treated with control shRNA or shRNA against Beclin1, or treated with BafA1 (100 nM, 6 h), followed by IB with the indicated antibodies. *n* = 3 independent experiments.

Scale bars, 10 μm. Data in a, b, e, and g-j are from one experiment that is representative of three independent experiments. For all quantification, data represents the mean ± SD derived from the indicated number of independent experiments. Comparisons were made using Student's *t* test. *, $P < 0.05$; **, $P < 0.01$; ***, $P < 0.001$; n.s., not significant. See Supplementary Fig. 13 for uncropped data of **a**, **b, e**, **g**, **h**.

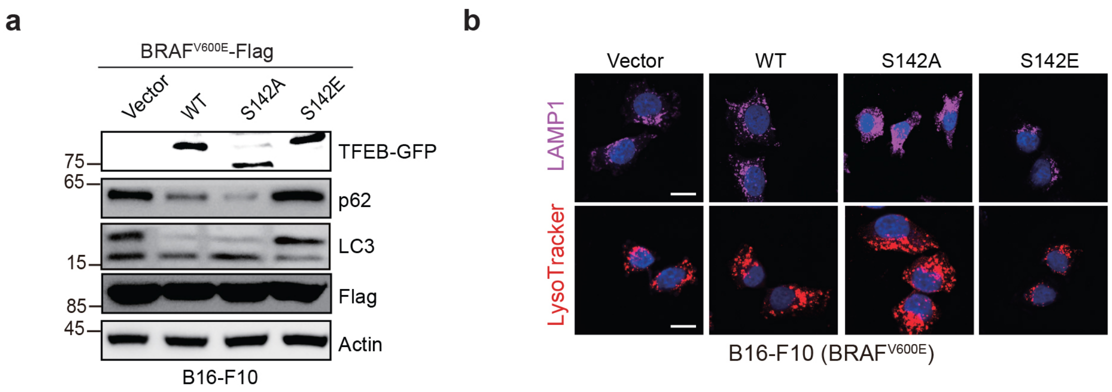

Supplementary Figure 8

Supplementary Figure 8. Effect of TFEB S142 phosphorylation on autophagy and lysosomal biogenesis in B16-F10 mouse melanoma cells expressing BRAF^{V600E}.

a Western blot analysis of autophagy (p62 and LC3) in B16 cells ectopically expressing BRAF^{V600E} along with WT TFEB or TFEB mutants. Actin served as a loading control. *n* = 4 independent experiments. See Supplementary Fig. 13 for uncropped data.

b Representative images of LAMP1 (purple) and LysoTracker Red (red) immunostaining of cells shown in (a). *n* = 3 independent experiments. Scale bars, 10 μm.

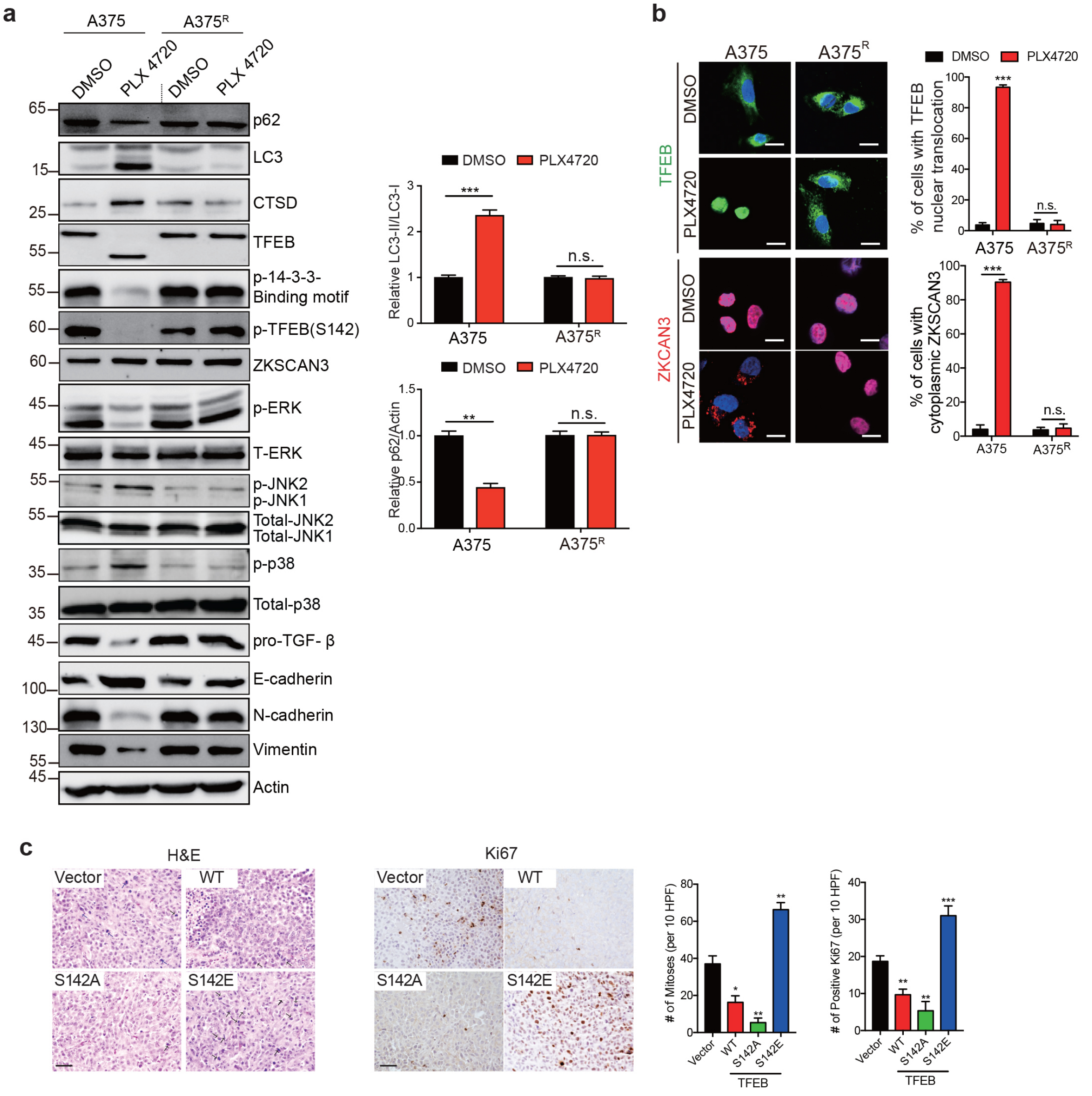

Supplementary Figure 9

Supplementary Figure 9. Role of TFEB S142 phosphorylation in tumor progression of A375 melanoma cells resistance to BRAFi.

a Immunoblot analyses of p62, LC3-I/II, Cathepsin D (CTSD), TFEB, phosphorylation of 14-3-3 proteins binding motif of TFEB, S142 phosphorylation, ZKSCAN3, EMT proteins (E-cadherin, N-cadherin, Vimentin), pro-TGF-β, and the activity of ERK, JNK1/2, p38 in A375 and A375^R cells in the presence and absence of PLX4720 (1 μM, 12 h). *n* = 3 independent experiments. Densitometric quantifications of the LC3-II/LC3-I (top) and p62/actin ratios (bottom) under the

indicated conditions are shown (right panels). *n* =3 independent experiments. See Supplementary Fig. 13 for uncropped data.

b Representative confocal images showing subcellular distribution of TFEB and ZKSCAN3 in A375 and A375^R cells in response to DMSO or PLX4720 treatment (1 μM, 12 h). The percentage of cells with nuclear translocation of TFEB and cytoplasmic distribution of ZKSCAN3 is quantified (right panels). *n* = 3 independent experiments. Scale bars, 10 μm.

(c) Representative images of H&E-stained section (left) and Ki67 (right) staining of the indicated A375^R xenograft tumor genotype. Arrows indicate mitotic cells. The levels of mitotic figures and Ki67$^+$ staining in indicated xenograft tumor genotype are quantified (right). Scale bars, 100 μm.

Data in a is from one experiment that is representative of three independent experiments. For all quantification, data represents the mean ± SD derived from indicated number of independent experiments. Comparisons were made using Student's *t* test. **, $P < 0.01$; ***, $P < 0.001$; n.s., not significant.

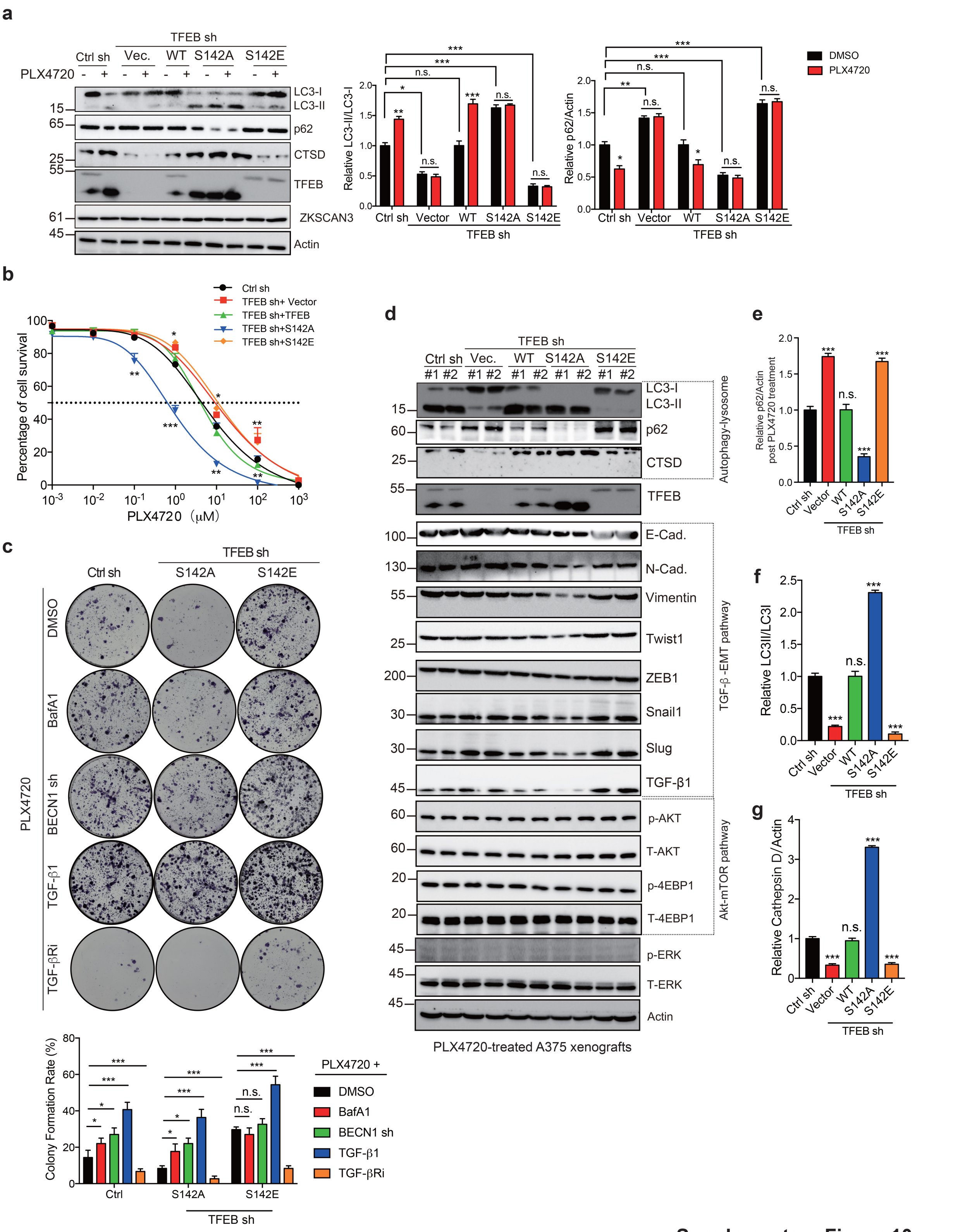

Supplementary Figure 10

Supplementary Figure 10. Role of TFEB S142 phosphorylation in $BRAF^{V600E}$ melanoma cell resistance to BRAF*i*.

a Immunoblot analysis (left) of autophagy (LC3 and p62) and lysosome (CTSD) activity in the indicated A375 cells in the presence or absence of PLX4720 treatment (1 μM, 12 h). Expression of TFEB and ZKSCAN3 is also shown. Densitometric quantifications of the LC3-II/LC3-I and p62/actin ratios under the indicated conditions are shown (right panels). $n = 3$ independent experiments.

b Viability of the indicated A375 cells after treatment with varying concentrations of PLX4720 for 2 days. $n = 3$ independent experiments.

c Colony formation assay of A375 cells expressing empty vector, $TFEB^{S142A}$, or $TFEB^{S142E}$ treated with PLX4720 (1 μM, 12 h) in the presence or absence of BafA1 (100 nM), Beclin1-specific shRNA, TGF-β1 (10 ng/ml), or TGF-β receptor inhibitor (TGF-βRi) SB431542 (10 μM) as indicated. Bars are the mean ± SD percentage of colonies for each group after 14 days (bottom). $n = 3$ independent experiments.

d Immunoblot analysis of autophagy (LC3 and p62) and lysosome (CTSD), EMT (E-cadherin, N-cadherin, Vimentin), EMT-TFs (Twist1, ZEB1, Snail, Slug), TGF-β, AKT, mTORC1, and ERK activities in the indicated PLX4720-treated A375 xenograft tumor genotypes (two randomly chosen samples per group; similar results observed in all samples per group).

(**e-g**) Densitometric quantifications of the LC3-II/LC3-I (**e**), p62/actin (**f**), and CTSD/actin ratios **g** in the indicated A375 xenograft tumor genotypes. $n = 3$ independent experiments.

Data in a and d are from one experiment that is representative of three independent experiments. For all quantification, data represents the mean ± SD derived from the indicated number of independent experiments. Comparisons were made using Student's *t* test. *, $P < 0.05$; **, $P < 0.01$; ***, $P < 0.001$; n.s., not significant. See Supplementary Fig. 13 for uncropped data of **a** and **d**.

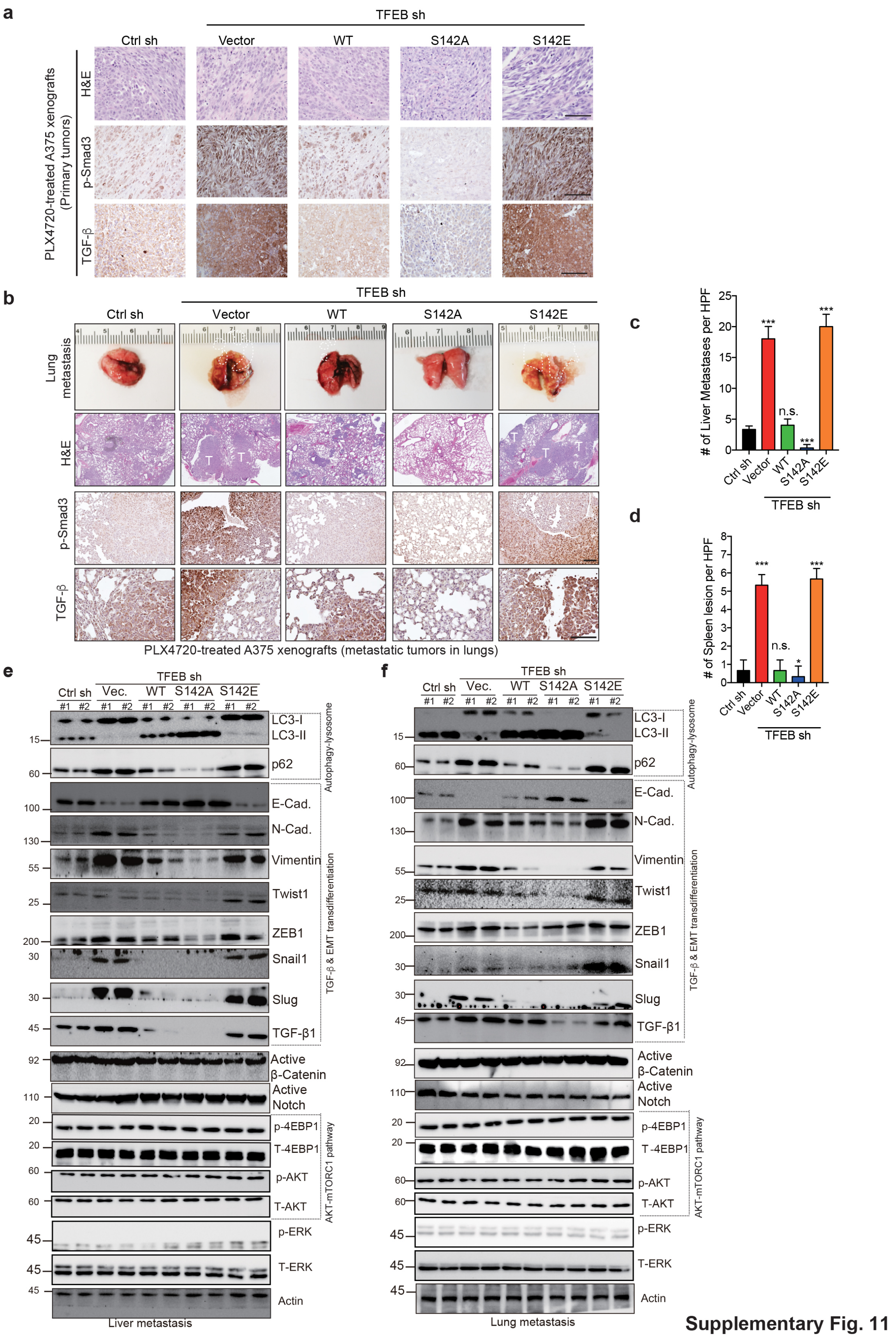

Supplementary Fig. 11

Supplementary Figure 11. TFEB inactivation promotes tumor metastasis in BRAF*i*-treated A375 xenografts.

a Representative H&E-stained sections and immunohistochemical (IHC) staining of TGF-β and p-Smad3 in the indicated A375 xenograft tumor genotypes. Data are from one animal that is representative of 5-6 animals in each group.

b TFEB S142 phosphorylation enhances tumor metastasis in mice inoculated with the indicated A375 xenograft genotypes. Representative gross images of lungs with metastatic nodules (upper panel) and H&E-stained sections and immunohistochemical analysis of TGF-β and p-Smad3 of metastasis nodules (bottom) are shown. Data are from one animal that is representative of 5-6 animals in each group.

c,d The number of metastatic colonization in lungs (**c**) and the spleen (**d**) was quantified. (n = 5-6 mice per group; data represents the mean ± SD).

e,f Immunoblot analysis of autophagy (p62 and LC3I/II), EMT, EMT-TFs, TGF-β, active β-catenin and Notch1 activity, and the AKT, mTORC1, and ERK activities in liver (**e**) and lung (**f**) metastatic nodules of the indicated xenograft genotype treated with PLX4720 (20 mg kg^{-1}) daily for 21 days (two randomly chosen samples per group; similar results observed in all samples per group). n = 3 independent experiments.

Scale bars, 100 μm. For all quantification, data represents the mean ± SD derived from the indicated number of independent experiments. Comparisons were made using Student's t test. *, $P < 0.05$; **, $P < 0.01$; ***, $P < 0.001$; n.s., not significant. See Supplementary Fig. 13 for uncropped data of **e** and **f**.

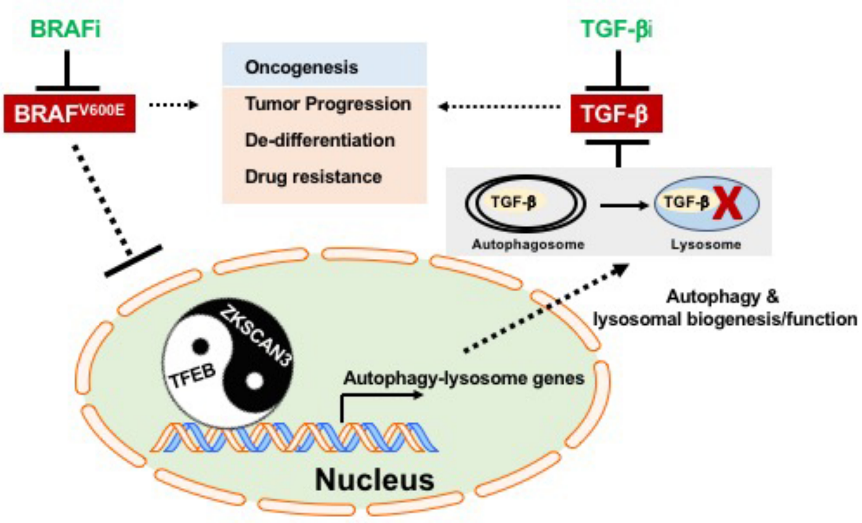

Supplementary Fig. 12

Supplementary Figure 12. A hypothetical model of TFEB/ZKSCAN3-mediated autophagy-lysosomal activation in BRAF^{V600E} melanoma progression and resistance to BRAF-targeted therapy. BRAF^{V600E} regulates the transcriptional activity of TFEB and ZKSCAN3 through ERK- and JNK2/p38 MAPK-dependent mechanisms, respectively, resulting in decreased net production of autophagy-lysosome-relevant factors, which can be reversed by BRAF inhibitors (BRAFi). Inhibition of BRAFi-induced transcriptional activation of the autophagy-lysosomal pathway restricts the lysosomal turnover of TGF-β and consequently activates TGF-β signaling, which promotes melanoma progression, tumor de-differentiation, metastasis, and resistance to BRAFi treatment. Inhibition of TGF-β signaling by TGF-β inhibitor (TGF-βi) re-sensitizes melanomas to BRAF-targeted therapy.

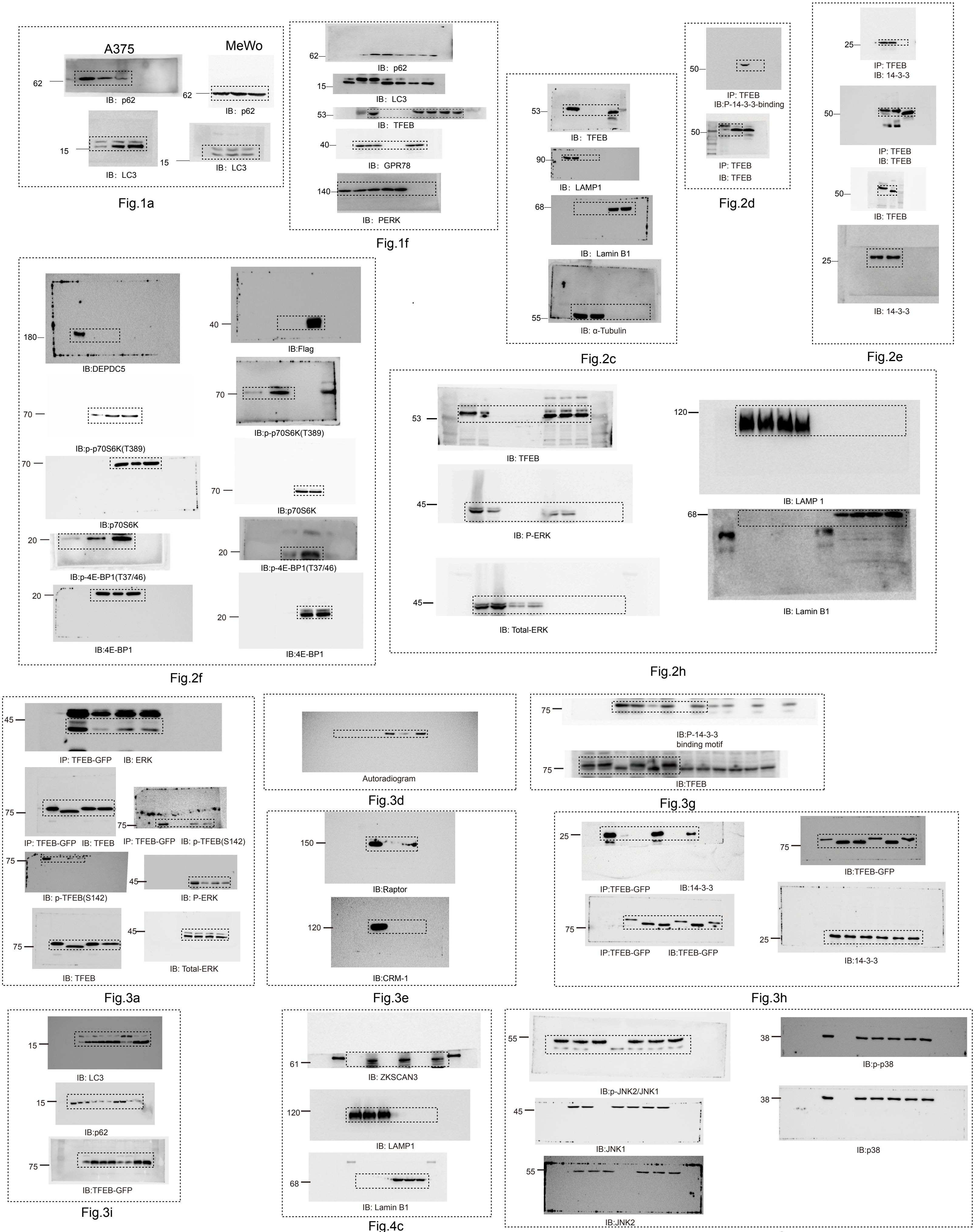

Fig.4f

Supplementary Fig. 13

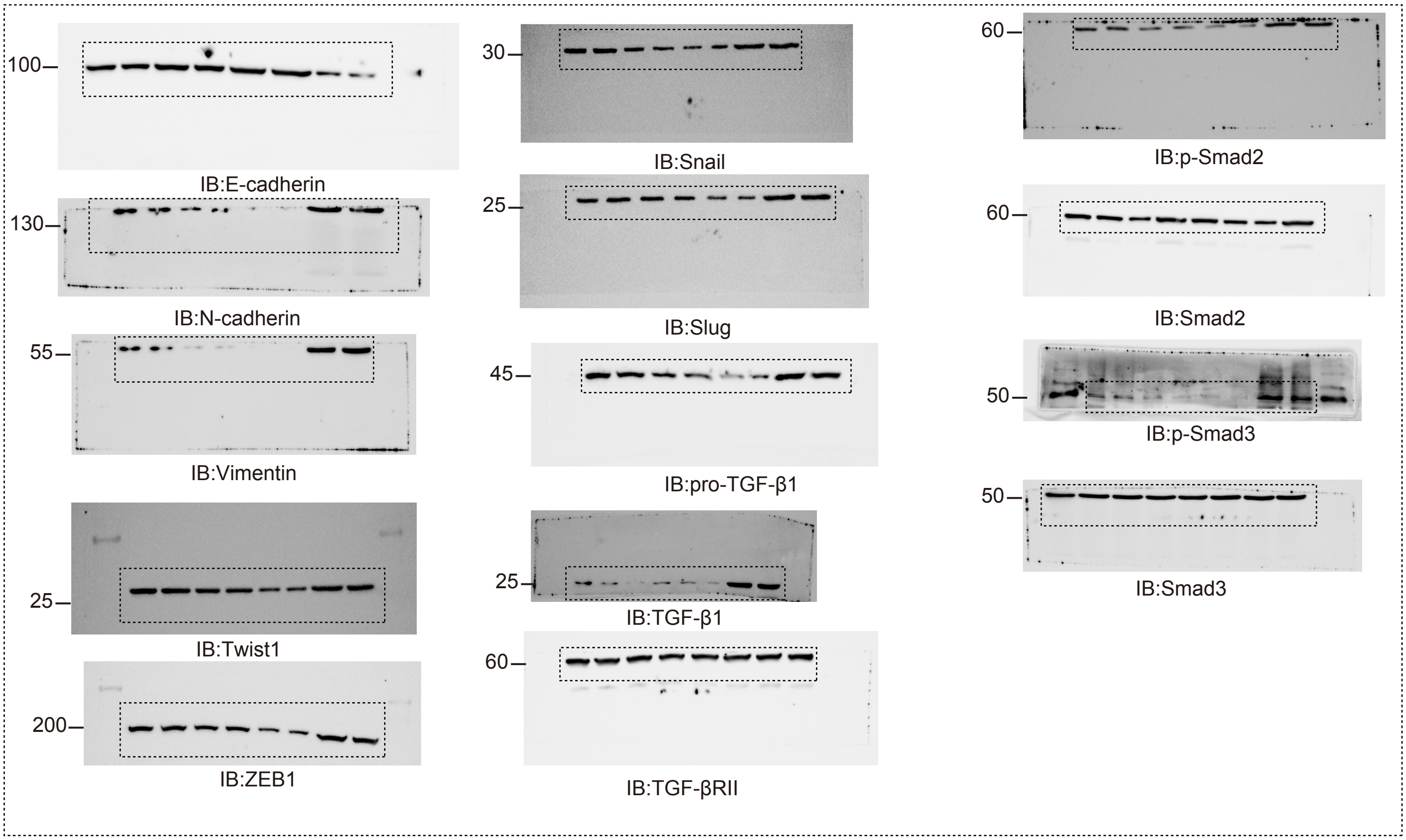

Fig.5f

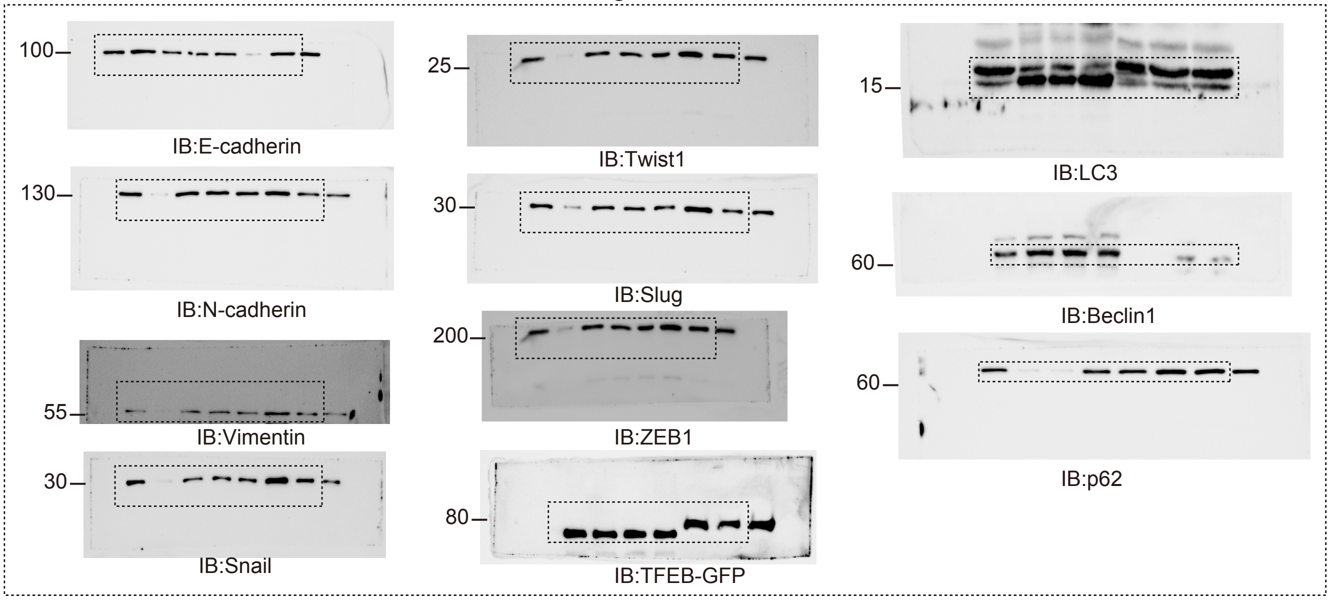

Fig.5i

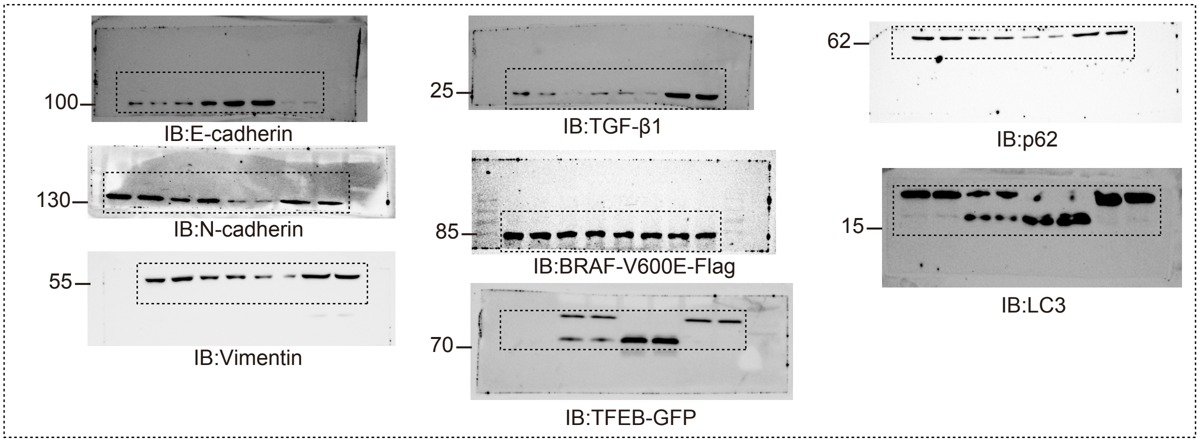

Fig.6c

Supplementary Fig. 13 continued

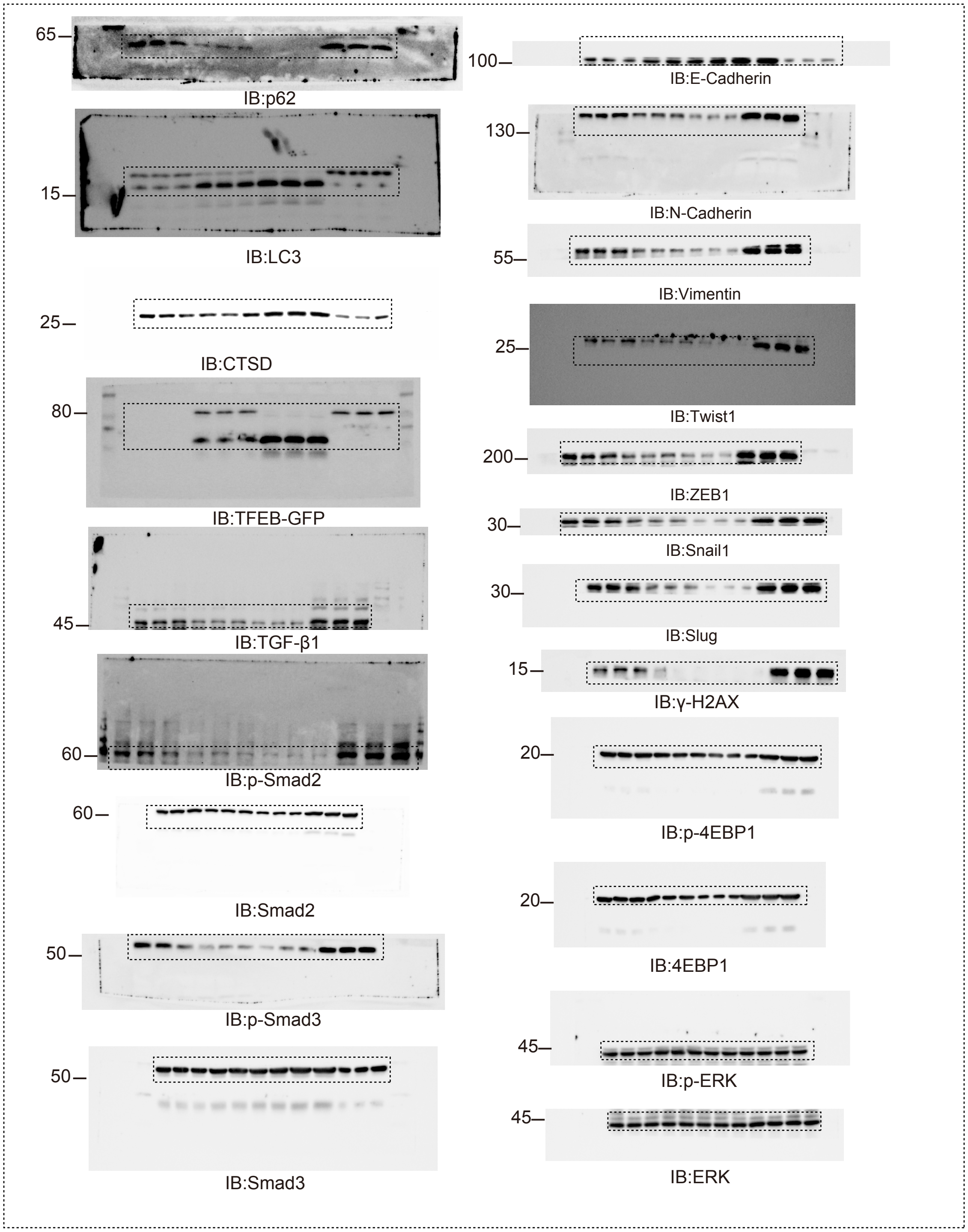

Fig.7e

Supplementary Fig. 13 continued

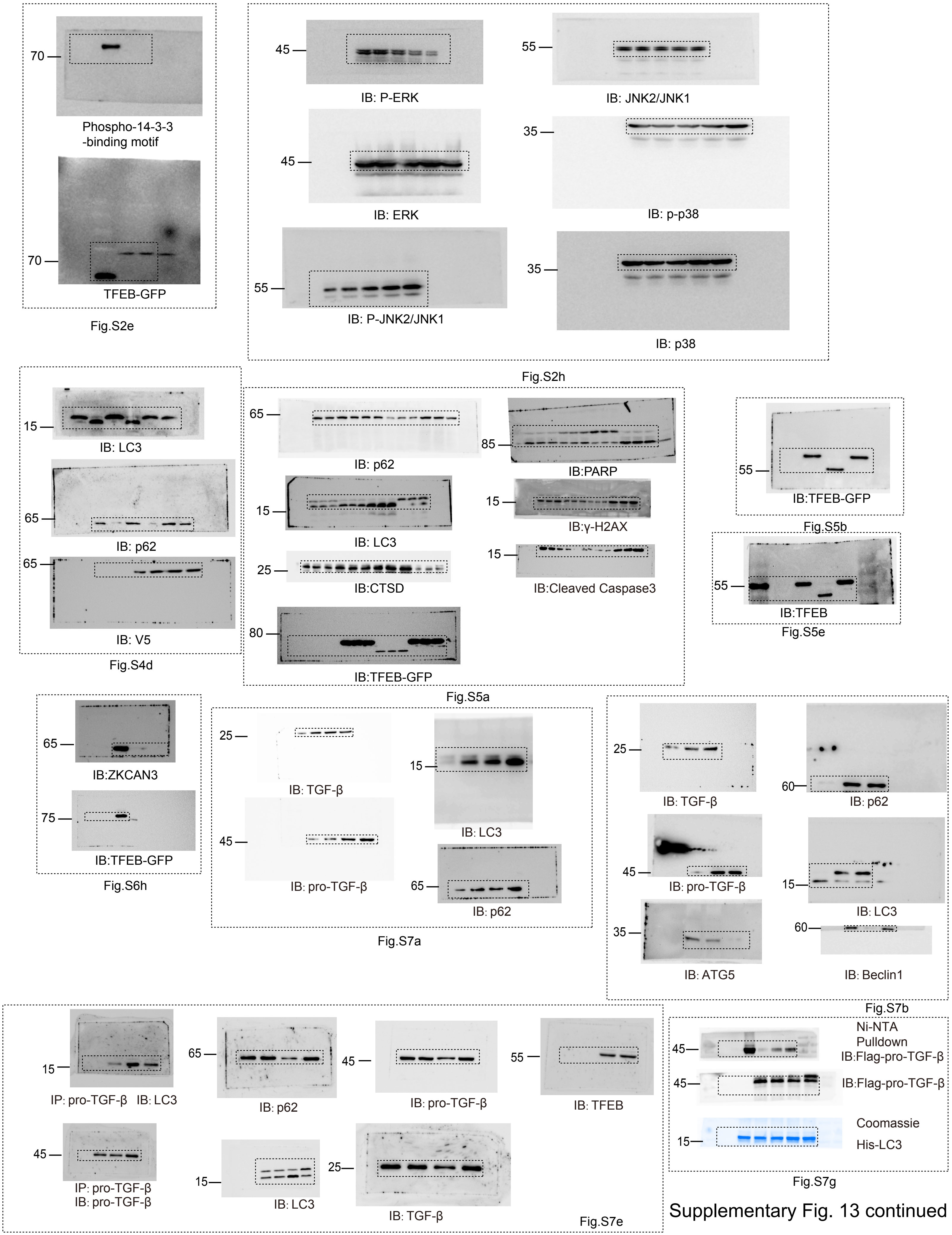

Supplementary Fig. 13 continued

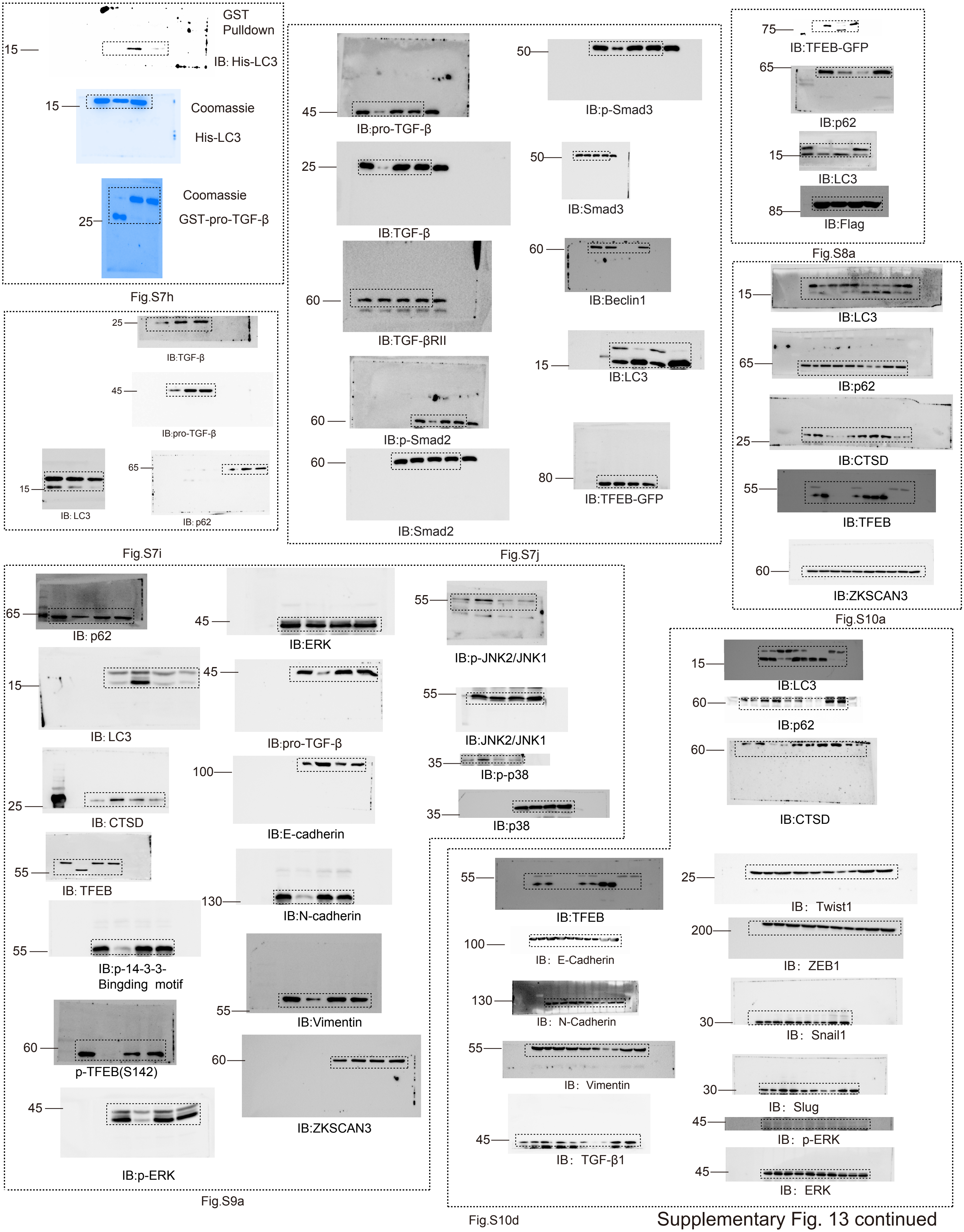

Supplementary Fig. 13 continued
