# Supplementary material for: Transcriptional regulation of autophagy-lysosomal function in BRAF-driven melanoma progression and chemoresistance
Source: Nat Commun. 2019 Apr 12;10:1693. doi: 10.1038/s41467-019-09634-8 (PMC6461621; doi:10.1038/s41467-019-09634-8)
Supplement: Supplementary file 3 — Description of Additional Supplementary Files [file 41467_2019_9634_MOESM3_ESM.pdf]

Description of Additional Supplementary Files

File Name: Supplementary Data 1

Description: Gene lists of the expression analysis in Figure 5 and Supplementary Figure 6.

- **(a)** Related to Fig. 5g. The autophagy-lysosome-related genes that were significantly upregulated by overexpression of $TFEB^{S142A}$ but downregulated by overexpression of $TFEB^{S142E}$ when compared with the vector control in A375 xenograft tumors (three replicates per group). FDR, P < 0.01; NES = 3.5.
- **(b)** Related to Supplementary Fig. 6c. Listed are genes involved in melanocyte differentiation that were differentially expressed in A375 xenograft tumors expressing WT TFEB or its mutant derivative.
- **(c)** Related to Supplementary Fig. 6d. EMT signature gene list showing differential expression in A375 xenograft tumors with the indicated genotypes.
- **(d)** Related to Fig. 5h. List of TGF-b target genes that are significantly upregulated in tumors expressing $TFEB^{S142E}$ but downregulated in tumors expressing $TFEB^{S142A}$.
- **(e)** Related to Supplementary Fig. 6e. Listed are overlapping genes that are upregulated in $TFEB^{S142E}$-expressing tumors and TGF-b signature.
- **(f)** Related to Supplementary Fig. 6e. Listed are overlapping genes that are downregulated in $TFEB^{S142A}$-expressing tumors and TGF-b signature.

File Name: Supplementary Data 2

Description: List of primers used for quantitative PCR.
