# Supplementary material for: Transcriptional regulation of autophagy-lysosomal function in BRAF-driven melanoma progression and chemoresistance
Source: Nat Commun. 2019 Apr 12;10:1693. doi: 10.1038/s41467-019-09634-8 (PMC6461621; doi:10.1038/s41467-019-09634-8)
Supplement: Supplementary file 6 — Reporting Summary [file 41467_2019_9634_MOESM6_ESM.pdf]

nature research

Corresponding author(s): Chengyu Liang

Last updated by author(s): 1-30-2019

Reporting Summary

Nature Research wishes to improve the reproducibility of the work that we publish. This form provides structure for consistency and transparency in reporting. For further information on Nature Research policies, see Authors & Referees and the Editorial Policy Checklist.

Statistics

For all statistical analyses, confirm that the following items are present in the figure legend, table legend, main text, or Methods section.

n/a	Confirmed	
☐	☒	The exact sample size (*n*) for each experimental group/condition, given as a discrete number and unit of measurement
☐	☒	A statement on whether measurements were taken from distinct samples or whether the same sample was measured repeatedly
☐	☒	The statistical test(s) used AND whether they are one- or two-sided<br>*Only common tests should be described solely by name; describe more complex techniques in the Methods section.*
☒	☐	A description of all covariates tested
☒	☐	A description of any assumptions or corrections, such as tests of normality and adjustment for multiple comparisons
☐	☒	A full description of the statistical parameters including central tendency (e.g. means) or other basic estimates (e.g. regression coefficient) AND variation (e.g. standard deviation) or associated estimates of uncertainty (e.g. confidence intervals)
☐	☒	For null hypothesis testing, the test statistic (e.g. *F*, *t*, *r*) with confidence intervals, effect sizes, degrees of freedom and *P* value noted<br>*Give P values as exact values whenever suitable.*
☒	☐	For Bayesian analysis, information on the choice of priors and Markov chain Monte Carlo settings
☒	☐	For hierarchical and complex designs, identification of the appropriate level for tests and full reporting of outcomes
☐	☒	Estimates of effect sizes (e.g. Cohen's *d*, Pearson's *r*), indicating how they were calculated

Our web collection on statistics for biologists contains articles on many of the points above.

Software and code

Policy information about availability of computer code

Data collection	No software was used
Data analysis	GraphPad Prism 7.0 (GraphPad Software, Inc.) was used to present data in graphs and for the statistical analyses of the data.<br>R was used for hierarchical clustering, principal component analysis, and RNAseq statistical analysis.<br>Living image 3.0 was used for live animal imaging analysis.<br>NIS-Elements AR software was used for imaging analysis of confocal microscopy.

For manuscripts utilizing custom algorithms or software that are central to the research but not yet described in published literature, software must be made available to editors/reviewers. We strongly encourage code deposition in a community repository (e.g. GitHub). See the Nature Research guidelines for submitting code & software for further information.

Data

Policy information about availability of data

All manuscripts must include a data availability statement. This statement should provide the following information, where applicable:

- Accession codes, unique identifiers, or web links for publicly available datasets
- A list of figures that have associated raw data
- A description of any restrictions on data availability

Generated plasmids and cell lines are available from the corresponding author upon request. The RNA-seq data is deposited into Gene Expression Omnibus (GEO) with the accession code GSE122614. All other data that support the findings of this study are available from the corresponding author on reasonable request.

Field-specific reporting

Please select the one below that is the best fit for your research. If you are not sure, read the appropriate sections before making your selection.

☒ Life sciences ☐ Behavioural & social sciences ☐ Ecological, evolutionary & environmental sciences

For a reference copy of the document with all sections, see nature.com/documents/nr-reporting-summary-flat.pdf

Life sciences study design

All studies must disclose on these points even when the disclosure is negative.

Sample size	We used a minimum of triplicates for each experiment with the control and the experimental samples to assess statistical significance. With 3 replicates for each condition, we will achieve 80% power to detect a minimum mean difference of 3 standard deviations between groups by two-sample t-test at alpha=0.05. For animal experiments, with 10 mice per group we will have 80% power to detect a difference of 0.46 in the outcome proportions between the control and the experimental groups at alpha=0.05.
Data exclusions	no data were excluded
Replication	We used a minimum of triplicates for each experiment and all attempts at replication were successful
Randomization	For image quantification, approximately 200 cells, randomly chosen from 10 high power fields and pooled from three independent experiments, were evaluated for the distribution pattern of the indicated molecules.<br>For melanoma response to the BRAF inhibitor, once tumors grew to a palpable size, mice of certain genotype were randomly chosen for PLX4720 treatment.
Blinding	All data acquisition and analysis were performed by investigators blinded to experimental group.

Reporting for specific materials, systems and methods

We require information from authors about some types of materials, experimental systems and methods used in many studies. Here, indicate whether each material, system or method listed is relevant to your study. If you are not sure if a list item applies to your research, read the appropriate section before selecting a response.

Materials & experimental systems

n/a	Involved in the study	
☐	☒	Antibodies
☐	☒	Eukaryotic cell lines
☒	☐	Palaeontology
☐	☒	Animals and other organisms
☒	☐	Human research participants
☒	☐	Clinical data

Methods

n/a	Involved in the study	
☒	☐	ChIP-seq
☒	☐	Flow cytometry
☒	☐	MRI-based neuroimaging

Antibodies

Antibodies used	Detailed information of all antibodies used in the study have been provided in the Method
Validation	All antibodies validation are available on the manufacturers' websites

Eukaryotic cell lines

Policy information about cell lines

Cell line source(s)	The sources of all cell line used in the work is stated
Authentication	All cell lines used in this work are directly obtained from ATCC with authentication, and cultured strictly following the provider's instructions.
Mycoplasma contamination	All cell lines were tested and confirmed to be free of mycoplasma
Commonly misidentified lines (See ICLAC register)	No commonly misidentified cell lines were used.

Animals and other organisms

Policy information about studies involving animals; ARRIVE guidelines recommended for reporting animal research

Laboratory animals	Detailed information of mouse strain, sex and age in animal experiments are included in the Method of the manuscript
Wild animals	N/A
Field-collected samples	N/A
Ethics oversight	None

Note that full information on the approval of the study protocol must also be provided in the manuscript.
